# Sex differences in the association between body mass index and quality of life among Korean older adults; evidence from a Community Health Survey in South Korea

**DOI:** 10.1186/s12877-024-05631-9

**Published:** 2024-12-26

**Authors:** Jieun Kim, Hooyeon Lee

**Affiliations:** 1 Department of Public Health, Graduate School, The Catholic University of Korea, Seoul, Korea; 2 Department of Preventive Medicine, College of Medicine, The Catholic University of Korea, Seoul, Korea

**Keywords:** Health-related quality of life, EQ-5D, BMI, Older adults

## Abstract

**Background:**

The high prevalence of underweight individuals is an important issue that has become increasingly common. Therefore, this study investigated the association between body mass index (BMI) and health-related quality of life (HRQoL) among Korean older adults using a nationwide population-based survey.

**Methods:**

Data from the 2021 Community Health Survey were used for this study. The study population was a total of 70,700 respondents. HRQoL was assessed using the EuroQoL health-related quality of life scale. Multiple logistic regression was applied to analyze the ORs for moderate or severe problems in the five EQ-5D dimensions. In addition, we performed multiple linear regression to identify the association between the total EQ-5D score and BMI after adjusting for age, marital status, income, education, health behaviors, and the presence of diabetes or hypertension.

**Results:**

Of the participants, 4.3% were underweight (3.3% of men and 5.1% of women). Being underweight is associated with poor HRQoL in both men and women. The relationship between obesity and HRQoL varied by sex. Men with pre-obesity and obesity were less likely to have “moderate or severe” problems in all EQ-5D dimensions, excluding mobility. However, women with obesity were more likely to have “moderate or severe” problems across EQ-5D dimensions, excluding anxiety/depression.

**Conclusions:**

Being underweight is associated with poor HRQoL among Korean older adults. Policy attention must be directed toward maintaining proper weight and promoting nutritional health at older ages, given that the number of older adults is expected to continue to increase.

## Background

The number of people aged 60 years or older worldwide is expected to increase from 1 in 8 by 2017 to 1 in 5 by 2050, with developing nations expected to face the highest rate of population aging [1]. In 2017, South Korea officially became an aged society, with more than 14% of its citizens aged 65 years or older [2]; this proportion is expected to reach 39.8% of the total population by 2049 [3].

Health-related quality of life (HRQoL) assesses health conditions’ impact on individuals’ daily lives. It is useful for describing the life experiences of older adults as they relate to their health [4–6]. Several determinants of HRQoL in older adults have been identified, including sex, age, marital status, income, education level, social support, history of falls, and chronic diseases [7–10]. Additionally, overweight and obesity are significantly associated with increasing burdens in obesity-related comorbidities, HRQoL, mobility, productivity, and disability [11].

Obesity in older adults is associated with poor physical and mental health outcomes, including a suboptimal health-related quality of life [12]. Older adults with obesity reported poorer physical HRQoL but better mental HRQoL [13]. Recently, there has been increasing evidence that patients, especially older adults, with elevated body mass index (BMI) may demonstrate lower all-cause and cardiovascular mortality than patients of normal weight [14, 15]. Meanwhile, older adults who are underweight have poor HRQoL regardless of compliance physical activity guideline status [16]. Being underweight is linked to poor quality of life among Japanese [17], and Canadian [18] older adults. A cohort study on older adults in South Korea reported that a lower BMI is associated with increased mortality risk, whereas a high BMI was not associated with increased mortality [19]. Indeed, deviations from the normal weight range are associated with lower HRQoL [20–23]. Underweight and obesity individuals reported impaired HRQoL particularly regarding worse physical functioning and physical well-being [15]. These findings highlight the importance of maintaining a healthy weight and avoiding nutritional risks at advanced ages.

Increased life expectancy will lead to growing numbers of frail and disabled older persons with a decreased HRQoL [24]. Although the impact of obesity on HRQoL is stronger in young people without co-morbidities, it progressively attenuates with advancing age when co-morbid conditions are present [25]. The association between BMI and disease may differ by geographical region, even among Asians [26]. Most previous studies have been from Western countries with a high prevalence of overweight and obesity.

The high prevalence of underweight individuals is important and has become more prevalent in recent years as the Korean population ages rapidly. Therefore, this study investigates the association between BMI and HRQoL among Korean older adults based on national population-based data, considering a sex-based differences in the BMI-HRQoL association [4].

## Methods

### Data source and study population

Data were analyzed using the 2021 Community Health Survey (CHS) of the Regional Public Health Act in South Korea [27]. The CHS is a nationwide population-based survey conducted annually since 2008 by the Korea Disease Control and Prevention Agency (KDCA) targeting adults aged ≥ 19 years. In the CHS, stratified cluster sampling and the systematic sampling method were used to select sample areas and households, respectively [28]. Trained surveyors visited each household and conducted face-to-face computer-assisted personal interviews. The surveyors attempted to prevent the spread of Coronavirus disease (COVID-19) during the interviews, which were conducted from August 16 to October 31, 2021 [29]. Of the 74,492 participants aged 65 or older, individuals with missing data (3,792(5.1%)) were excluded. The final study population comprised 70,700 participants.

### Dependent variable

HRQoL was assessed using the EuroQoL health-related quality of life scale (EQ-5D-3 L), the most widely used generic index measures of HRQoL [30]. Five health dimensions were assessed using the EQ-5D questionnaire: mobility, self-care, usual activities, pain/discomfort, and anxiety/depression. For each dimension, three levels were applied to describe severity: have no problems, have moderate problems, and have severe problems. These levels could describe 243 different health states [30]. The EQ-5D index score, converted from EQ-5D-3 L, was calculated based on the South Korea values set by the Korea Disease Control and Prevention Agency [31]. The maximum score for this value is 1, and a score close to 1 indicates better health. The Korean version of the EQ-5D has been proven valid and reliable [32].

### Independent variable

BMI was used as an independent variable of interest, which was calculated as self-reported current weight (kg) / [height (m)]^2^ based on the Obesity Guideline by the World Health Organization for the Asia Pacific Region and the Korean Society for the Study of Obesity (KSSO). Participants were divided into four groups according to their BMI values: Underweight (< 18.5 kg/m^2^), Normal (18.5–22.9 kg/m^2^), Pre-obesity (23.0–24.9 kg/m^2^), and Obesity (≥ 25.0 kg/m^2^) [33, 34].

### Covariates

Age was categorized into four groups; 65–69 years, 70–74 years, 75–79 years, ≥ 80 years. Annual household income was categorized based on USD into three groups: < $4,700, $4,700–$15,664, and > $15,664. An annual household income of $4,700 was the standard for livelihood benefits for one-person households in 2021 under the National Basic Living Security Act 8 in South Korea. Under this Act, an annual household income of $15,664 is the standard median income for one-person households [35]. Education was categorized into three groups: uneducated(including illiterate) or elementary school graduate, junior high school graduate or high school graduate, and college graduate or university graduate or higher.

Marital status was categorized into two groups: currently married and never married/divorced/widowed. The administrative district units in South Korea are divided into eup, myeon, and dong [36]. Residence area was classified as “eup/myeon” (rural area) and “dong” (urban area).

Smoking status was categorized into three groups: current (cigarette or e-cigarette), past, and never smoker. Participants were categorized into high-risk, moderate, and non-drinking groups. High-risk drinking was defined as drinking more than seven glasses of alcoholic drink for men and more than five glasses for women twice per week or more [37]. Moderate drinking was defined as drinking less than six glasses of alcoholic drink for men and less than four glasses for women twice per week or more, or regardless of the amount of alcohol consumed, drinking less than four times a month or less. The non-drinking group was classified as those that had not consumed any alcohol in the previous year. The frequency of breakfast consumption was categorized into two groups: 5–7/week and 0–4/week [38]. Walking was calculated as the activity during the last week, divided into “yes” (regular walking for at least 30 min 5 times/week in the past week) and “no” [39]. Diabetes or hypertension were classified based on whether there has been a diagnosis made by a physician: “yes” if they had either, or “no” if they did not have both.

### Statistical analyses

Sampling weights based on the sample design of the South Korean CHS were applied to the statistical analyses to present unbiased estimated representative data for the entire South Korean population. Chi-square tests and ANOVA were used to compare the EQ-5D index among the BMI groups. Multiple logistic regression was employed to analyze the ORs for moderate or severe problems across the five EQ-5D dimensions. In addition, a multiple linear regression analysis was performed to identify the association between EQ-5D scores and BMI after adjusting for age, marital status, income, education, health behaviors and, diabetes or hypertension.

Because there is a sex differences in the BMI-HRQoL association, all analyses above were performed separately by sex. All analyses were performed using SAS version 9.4 (SAS Institute Inc., Cary, NC, USA), and the statistical significance level was set at *P* < 0.05.

### Ethical considerations

The study design and survey contents were approved by Statistics South Korea (No. 117075). This study was not subject to deliberation by the research ethics committee as it was conducted directly or commissioned by the state or local government to review and evaluate public welfare or service programs (Enforcement Rule of Bioethics and Safety Act, Article 2).

## Results

Table 1 presents the participants’ sociodemographic characteristics, health behaviors, BMI, and diabetes and hypertension status. Of the 70,700 participants, 39,946(56.5%) were women, and 33.2% were aged 65–69 years. Among the participants, 39.9% were normal weight, while 4.3% and 27.7% were underweight and obesity, respectively. The proportion of underweight was 3.3% for men and 5.1% for women. Of the participants, 9.2% were current smoker, and 64.5% were never smoker.

Table 2 describes the distribution of experiences with ‘moderate or severe’ problems across all five EQ-5D dimensions and EQ-5D utility scores by BMI. Among men who were obesity and underweight, 0.6% and 3.4% reported severe problems in the mobility dimension, respectively. Among men and women who were underweight, 7.9% and 11.7% reported severe problems in the pain/discomfort dimension, respectively. Both women and men with underweight had severe problems in all five dimensions compared to other BMI categories. The EQ-5D index score was 0.83 for men with underweight and 0.79 for women with underweight.

**Table 1:** General characteristics of the participants(*N* = 70,700)

Variables	Men (*n* = 30,754)	Women (*n* = 39,946)	Total (*n* = 70,700)
	%	%	%
Age (years)			
65–69	34.3	32.3	33.2
70–74	26.7	25.7	26.1
75–79	20.0	19.6	19.8
≥ 80	19.0	22.3	20.8
Marital status			
Currently married	83.5	49.8	65.2
Never married, Separated, Divorced, Widowed	16.5	50.2	34.8
Annual household income (USD)			
> 15,664	56.3	47.0	51.3
4,700–15,664	35.9	39.2	37.7
< 4,700	7.8	13.7	11.0
Education			
≥ College graduate	19.6	6.5	12.5
High school, Junior high school graduate	51.4	34.6	42.3
≤ Elementary school graduate	29.0	58.9	45.3
Residence area			
Rural	26.6	26.2	26.4
Urban (dong)	73.4	73.8	73.6
Smoking status			
Current smoker	18.3	1.6	9.2
Past smoker	55.7	1.6	26.3
Never smoker	26.0	96.7	64.5
Drinking status			
High-risk drinking	10.5	0.5	4.9
Moderate drinking	66.3	27.3	44.3
Non-drinking	23.3	72.2	50.9
Breakfast			
0–4/week	7.6	9.4	8.6
5–7/week	92.4	90.6	91.4
Regular walking			
≥ 5 day/week (30 min/day)	50.9	43.5	46.9
< 5 day/week (30 min/day)	49.1	56.5	53.1
Body mass index			
< 18.5 (Underweight)	3.3	5.1	4.3
18.5–22.9 (Normal)	39.1	40.7	39.9
23.0–24.9 (Pre-obesity)	31.0	25.6	28.1
≥ 25.0 (Obesity)	26.6	28.7	27.7
Diabetes or Hypertension			
Yes (≥ 1)	59.3	62.1	60.8
No	40.7	37.9	39.2

**Table 2:** Health-related quality(EQ-5D) of life according to BMI categorizations

Variables	Men				Women			
	Normal (*n* = 12,147)	Underweight (*n* = 1,208)	Pre-obesity (*n* = 9,249)	Obesity (*n* = 8,150)	Normal (*n* = 17,048)	Underweight (*n* = 2,360)	Pre-obesity (*n* = 9,725)	Obesity (*n* = 10,813)
	%	%	%	%	%	%	%	%
Mobility								
No problems	76.5	56.5	81.4	77.7	63.8	50.0	64.4	55.3
Moderate problems	22.2	40.1	18.1	21.7	34.7	45.9	34.7	43.5
Severe problems	1.2	3.4	0.5	0.6	1.5	4.1	0.9	1.2
*p-value*	< 0.0001				< 0.0001			
Self-care								
No problems	90.7	79.7	93.9	92.7	86.6	75.0	88.7	86.4
Moderate problems	7.9	16.2	5.5	6.2	11.6	20.2	10.2	12.3
Severe problems	1.4	4.1	0.6	1.0	1.8	4.8	1.1	1.3
*p-value*	< 0.0001				< 0.0001			
Usual activities								
No problems	82.2	64.3	87.0	85.2	73.0	58.2	74.9	70.4
Moderate problems	15.9	30.3	12.0	13.6	24.8	36.0	23.9	27.5
Severe problems	1.9	5.4	1.0	1.2	2.2	5.7	1.2	2.1
*p-value*	< 0.0001				< 0.0001			
Pain/Discomfort								
No problems	61.3	47.0	66.7	62.1	41.0	31.5	40.8	35.9
Moderate problems	34.9	45.1	30.6	34.6	51.8	56.9	52.5	56.1
Severe problems	3.8	7.9	2.6	3.3	7.2	11.7	6.7	8.0
*p-value*	< 0.0001				< 0.0001			
Anxiety/Depression								
No problems	84.7	73.2	87.5	87.0	74.9	64.5	75.7	75.0
Moderate problems	14.1	23.4	11.8	12.3	23.6	32.2	22.8	23.4
Severe problems	1.3	3.3	0.7	0.7	1.5	3.3	1.4	1.6
*p-value*	< 0.0001				< 0.0001			
EQ-5D index*	0.90	0.83	0.93	0.91	0.85	0.79	0.86	0.84
*p-value*	< 0.0001				< 0.0001			

* ANOVA analysis

**Fig. 1:**
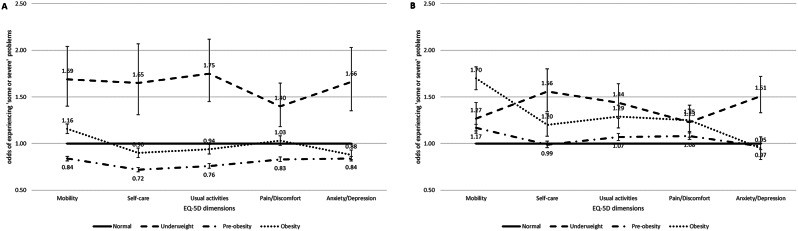
Adjusted odds of experiencing ‘moderate or severe’ problems across EQ-5D dimensions(mobility, self-care, usual activities, pain/discomfort, anxiety/depression) by BMI groups for 30,754 men (**A**) and 39,946 women (**B**), ≥ 65 years of age. Adjusted for age, marital status, annual household income, education, residence area, smoking status, drinking status, breakfast, regular walking and Diabetes or Hypertension

Table 3; Fig. 1 summarizes the findings of the logistic regression analyses of the reported problems in each of the five EQ-5D dimensions. In the adjusted model, compared with normal weight, men with underweight were more likely to have problems in mobility (OR 1.69, 95% CI 1.40–2.04), self-care (OR 1.65, 95% CI 1.31–2.07), usual activities (OR 1.75, 95% CI 1.45–2.12), pain/discomfort (OR 1.40, 95% CI 1.18–1.65), anxiety/depression (OR 1.66, 95% CI 1.35–2.03). For women, being underweight had increased odds of reporting moderate or severe problems in mobility (OR 1.27, 95% CI 1.11–1.44), self-care (OR 1.56, 95% CI 1.34–1.80), usual activities (OR 1.44, 95% CI 1.27–1.64), pain/discomfort (OR 1.23, 95% CI 1.07–1.41), anxiety/depression (OR 1.51, 95% CI 1.33–1.72). Men with underweight had the highest odds of reporting moderate or severe problems in all EQ-5D dimensions. Men and women who were pre-obesity had lower odds of reporting moderate or severe problems in all EQ-5D dimensions, excluding the mobility, anxiety/depression, pain/discomfort dimensions for women.

**Table 3:** Adjusted odds of experiencing ‘moderate or severe’ problems across EQ-5D dimensions

Variables	Mobility		Self-care		Usual activities		Pain/Discomfort		Anxiety/Depression	
	OR	95% CI	OR	95% CI	OR	95% CI	OR	95% CI	OR	95% CI
**Unadjusted**										
Men										
Normal	1.00	Reference	1.00	Reference	1.00	Reference	1.00	Reference	1.00	Reference
Underweight	2.52	2.13–2.98	2.49	2.02–3.07	2.57	2.17–3.04	1.79	1.52–2.10	2.02	1.67–2.45
Pre-obesity	0.75	0.69–0.81	0.64	0.56–0.72	0.69	0.63–0.76	0.79	0.74–0.85	0.79	0.72–0.87
Obesity	0.94	0.86–1.02	0.77	0.67–0.87	0.80	0.73–0.88	0.97	0.90–1.04	0.82	0.74–0.92
Women										
Normal	1.00	Reference	1.00	Reference	1.00	Reference	1.00	Reference	1.00	Reference
Underweight	1.76	1.56–1.98	2.15	1.87–2.47	1.94	1.72–2.19	1.52	1.33–1.72	1.65	1.46–1.87
Pre-obesity	0.97	0.91–1.04	0.82	0.74–0.91	0.91	0.84–0.97	1.01	0.94–1.08	0.96	0.89–1.04
Obesity	1.42	1.34–1.51	1.01	0.93–1.10	1.13	1.06–1.21	1.24	1.16–1.32	1.00	0.93–1.07
**Adjusted** ^*^										
Men										
Normal	1.00	Reference	1.00	Reference	1.00	Reference	1.00	Reference	1.00	Reference
Underweight	1.69	1.40–2.04	1.65	1.31–2.07	1.75	1.45–2.12	1.40	1.18–1.65	1.66	1.35–2.03
Pre-obesity	0.84	0.76–0.92	0.72	0.63–0.82	0.76	0.69–0.84	0.83	0.77–0.89	0.84	0.76–0.93
Obesity	1.16	1.05–1.27	0.90	0.79–1.04	0.94	0.84–1.04	1.03	0.95–1.11	0.88	0.79–0.98
Women										
Normal	1.00	Reference	1.00	Reference	1.00	Reference	1.00	Reference	1.00	Reference
Underweight	1.27	1.11–1.44	1.56	1.34–1.80	1.44	1.27–1.64	1.23	1.07–1.41	1.51	1.33–1.72
Pre-obesity	1.17	1.08–1.26	0.99	0.89–1.10	1.07	0.99–1.16	1.08	1.00-1.16	0.97	0.89–1.05
Obesity	1.70	1.58–1.83	1.20	1.09–1.32	1.29	1.20–1.40	1.25	1.17–1.34	0.95	0.88–1.02

* Multiple logistic regression model adjusted for: age, marital status, annual household income, education, residence area, smoking status, drinking status, breakfast, regular walking and Diabetes or Hypertension

Table 4 summarizes the total EQ-5D scores according to BMI, considering the sociodemographic characteristics, health behaviors, and diabetes and hypertension status of the participants. Age, marital status, income, education, residence area, breakfast, regular walking, and diabetes or hypertension status were significantly associated with EQ-5D scores in both men and women. Non-drinking was associated with lower EQ-5D scores than high-risk drinking. Compared to normal BMI, being underweight was significantly associated with decreased EQ-5D total scores for both men and women.

**Table 4:** Multiple linear regression exploring the relationship between BMI and EQ-5D index scores

Variables	Men (*n* = 30,754)		Women (*n* = 39,946)	
	Parameter estimate	*p*-value	Parameter estimate	*p*-value
Age (years)				
65–69	1		1	
70–74	-0.010	< 0.0001	-0.021	< 0.0001
75–79	-0.027	< 0.0001	-0.056	< 0.0001
≥ 80	-0.075	< 0.0001	-0.106	< 0.0001
Marital status				
Currently married	1		1	
Never married, Separated, Divorced, Widowed	-0.012	< 0.0001	-0.007	0.0001
Annual household income (USD)				
> 15,664	1		1	
4,700 to < 15,664	-0.020	< 0.0001	-0.011	< 0.0001
< 4,700	-0.040	< 0.0001	-0.035	< 0.0001
Education				
≥ College graduate	1		1	
High school, Junior high school graduate	-0.012	< 0.0001	-0.018	< 0.0001
≤ Elementary school graduate	-0.034	< 0.0001	-0.053	< 0.0001
Residence area				
Rural	1		1	
Urban (dong)	-0.007	< 0.0001	-0.011	< 0.0001
Smoking status				
Current smoker	1		1	
Past smoker	-0.005	0.0115	0.005	0.5455
Never smoker	0.005	0.0412	0.033	< 0.0001
Drinking status				
High-risk drinking	1		1	
Moderate drinking	-0.002	0.4748	-0.028	0.0380
Non-drinking	-0.038	< 0.0001	-0.054	< 0.0001
Breakfast				
0–4/week	1		1	
5–7/week	0.026	< 0.0001	0.035	< 0.0001
Regular walking				
≥ 5 day/week (30 min/day)	1		1	
< 5 day/week (30 min/day)	-0.044	< 0.0001	-0.056	< 0.0001
Body mass index				
18.5–22.9 (Normal)	1		1	
< 18.5 (Underweight)	-0.044	< 0.0001	-0.031	< 0.0001
23.0–24.9 (Pre-obesity)	0.013	< 0.0001	0.001	0.6025
≥ 25.0 (Obesity)	0.006	0.0022	-0.017	< 0.0001
Diabetes or Hypertension				
Yes (≥ 1)	1		1	
No	0.020	< 0.0001	0.020	< 0.0001

## Discussion

This study investigated the relationship between BMI and HRQoL in a nationally representative sample of Korean older adults. Underweight prevalence was 4.3% in Korean older adults aged 65 and older. The underweight prevalence rate was higher in women than in men (men: 3.3%, women: 5.1%). Among the participants, 27.7% were obesity (men: 26.6%, women: 28.7%). In Japan, the obesity prevalence among older adults was 28.7% and 20.2% in men and women, respectively, and the underweight prevalence was 5.2% and 7.7%, respectively [40]. Our study showed a lower underweight prevalence than a Japanese study. Compared to Japanese women, obesity was more prevalent among Korean women.

Compared with normal weight, both women and men with underweight had increased odds of reporting moderate or severe problems in all five EQ-5D dimensions. Moreover, being underweight was significantly associated with decreased EQ-5D total scores for both men and women compared to normal BMI. This is consistent with previous studies demonstrating that being underweight is associated with poor HRQoL [41, 42]. Men with underweight were most likely to have problems in the usual activities dimension and women with underweight were most likely to have problems in self-care dimension compared to normal weight.

The relationship between BMI and HRQoL in our study varied by sex, particularly for the population with obesity. When total EQ-5D index scores were considered after adjusting for sociodemographic characteristics and health behaviors, men who were pre-obesity and obesity exhibited higher EQ-5D index scores than those of normal weight. Additionally, men with pre-obesity and obesity were less likely to have “moderate or severe” problems in all EQ-5D dimensions, excluding mobility. Recent studies have reported an obesity paradox to HRQoL outcomes, suggesting that overweight and obesity may paradoxically correlate with higher HRQoL, called “obesity-HRQoL paradox” [4]. However, women with obesity were more likely to have “moderate or severe” problems across EQ-5D dimensions, excluding anxiety/depression.

A possible explanation for these sex-based differences in obesity was that women might be more susceptible to distress associated with body weight or body image than men [43], and obesity among men was associated with greater vitality and better HRQoL mental health [44]. It could also be explained by cultural perceptions about personal body weight and more discrimination against women with excess body weight in their work-related life and social roles [45].

Consistent with previous findings, this study showed that age and marital status were associated with EQ-5D scores. Physical inactivity negatively impacted HRQoL [46]. Those with lower annual household income and education levels had lower EQ-5D index score [10, 47, 48]. The higher the frequency of breakfast, the higher the HRQoL. This is consistent with previous findings that the higher the nutrition risk, the lower the HRQoL [49].

The adverse effects of smoking on health are well documented; however, its impact on HRQoL is not well known. Current smokers generally have lower HRQoL than never smokers. In addition, current smokers who had attempted to quit have lower HRQoL than those who have not attempted to quit [50]. Recent quitters have shown improvements in mental health compared to continuing or new smokers [51]. This study showed that among older men, never smoker reported higher EQ-5D scores than current smokers, and past smoker reported lower EQ-5D score than current smokers. A previous study in China reported a difference in this relationship among young, middle-aged, and older adults. Compared to never smokers, former smokers reported significantly lower EQ-5D-3 L utility values in middle-aged adults, while current smokers reported significantly higher EQ-5D-3 L utility values in older adults [52].

Men and women who were non-drinking have poor HRQoL. Moderate alcohol consumption in older adults was associated with better HRQoL than non-drinkers [53]. Both self-reported moderate and heavy drinkers reported better physical HRQoL [54]. However, differences in patterns of alcohol consumption across populations may account for the heterogeneity of these findings. Specifically, in addition to average alcohol consumption, the context of drinking, as well as the type of alcoholic beverage, may affect health outcomes. Frequent/moderate drinkers are more likely to have better self-rated health than non-drinkers [55].

Many developed countries are battling pre-obesity or obesity, and the high obesity prevalence in South Korea has also been a major public health problem. However, our study found that men with pre-obesity were less likely to have problems in all five EQ-5D dimensions, whereas men and women with underweight were more likely to have problems. A previous study has suggested that underweight older adults are expected to live the shortest lives and spend the fewest years in an active state [17]. In addition, pre-obesity is not associated with an increased mortality risk, while underweight has an increased mortality risk [56].

Disease prevalence is an important for the older adults, but health-related life satisfaction is also important. Older adults with underweight were more likely to have problems in all dimensions related to quality of life, as well as anxiety/depression. Unlike adolescents and adults, older adults should be more considered about being underweight. This is because underweight is negative for quality of life as well as physical health, such as sarcopenia [57].

In addition, the relationship between BMI and quality of life in older women in South Korea represents a U-shape. However, the lower the BMI, the more negative the quality of life in older men. In South Korea, where the population is rapidly aging, this should be considered in the public health. Policymakers should not only consider obesity but all groups other than normal weight. Underweight develops slowly, within both individuals and populations; it will also take time to establish new habits and build new structures to support a healthy diet. It should be considered to derive a healthy method to solve underweight in future studies so that appropriate interventions can be developed in the long term.

The association between BMI and HRQoL varied by sex in our study, particularly in the obese population. Paradoxically, overweight and obesity may correlate with higher HRQoL, especially in men. The findings of this study emphasize the importance of considering sex and BMI when assessing the relationship between BMI and HRQoL in older adults. This information can help in developing effective strategies for obesity counseling for older adults.

This study has some limitations. First, weight and height were self-reported, possibly leading to the underestimation of weight, overestimation of height, and underestimation of overall BMI [58]. Second, we did not consider all possible confounding factors, such as living arrangement, pain, osteoarthritis, and long-standing health conditions [59]. Lastly, we used cross-sectional survey data, and causal relationships between BMI and HRQoL could not be identified. However, this study used only nationally representative data from South Korea.

## Conclusion

Being underweight is associated with poor HRQoL among Korean older adults. The high prevalence and substantial loss of HRQoL associated with being underweight highlights the potential impact that interventions aimed at prevention or alleviation may have on the health of older adults. Policy attention must be directed toward maintaining proper weight and promoting nutritional health at an older age.

## Data Availability

The current study used data from the 2021 Community Health Survey in South Korea that are publicly available. The analytic materials are available at Korea Disease Control and Prevention Agency: https://chs.kdca.go.kr/chs/rdr/rdrInfoProcessMain.do. The current study was not a preregistered study.

## References

[1] World Population Ageing. 2017 Highlights. In. New York: United Nations; 2017.

[2] Baek JY, Lee E, Jung HW, Jang IY. Geriatrics fact sheet in Korea 2021. Ann Geriatr Med Res. 2021;25(2):65–71. 34187140 10.4235/agmr.21.0063 PMC8272996

[3] Population Projections for Korea. 2020–2070. In. Seoul: Staitstics Korea; 2022.

[4] Zhang J, Xu L, Li J, Sun L, Qin W, Ding G, Wang Q, Zhu J, Yu Z, Xie S, et al. Gender differences in the association between body mass index and health-related quality of life among adults:a cross-sectional study in Shandong, China. BMC Public Health. 2019;19(1):1021. 31366336 10.1186/s12889-019-7351-7 PMC6668122

[5] Malva JO, Bousquet J. Operational definition of active and healthy ageing: Roadmap from concept to change of management. Maturitas. 2016;84:3–4. 26704254 10.1016/j.maturitas.2015.11.004

[6] Ward M, McGarrigle CA, Kenny RA. More than health: quality of life trajectories among older adults-findings from the Irish longitudinal study of Ageing (TILDA). Qual Life Res. 2019;28(2):429–39. 30209723 10.1007/s11136-018-1997-y

[7] Prause W, Saletu B, Tribl GG, Rieder A, Rosenberger A, Bolitschek J, Holzinger B, Kapfhammer G, Katschnig H, Kunze M, et al. Effects of socio-demographic variables on health-related quality of life determined by the quality of life index–German version. Hum Psychopharmacol. 2005;20(5):359–65. 15981308 10.1002/hup.699

[8] Abu Hammattah A, Mohd Yunus R, Matthias Muller A, Bahyah Kamaruzzaman S, Naqiah Hairi N. Association between structural social support and quality of life among urban older malaysians. Australas J Ageing. 2021;40(4):390–6. 33594750 10.1111/ajag.12919

[9] Li PS, Hsieh CJ, Miao NF. A study of physical activity, Frailty, and Health-Related Quality of Life among Community-Dwelling older adults in Taiwan. J Nurs Res. 2020;28(6):e124. 32941303 10.1097/JNR.0000000000000402 PMC7664969

[10] Jia H, Lubetkin EI. The impact of obesity on health-related quality-of-life in the general adult US population. J Public Health (Oxf). 2005;27(2):156–64. 15820993 10.1093/pubmed/fdi025

[11] MacEwan JP, Chiu K, Ahmad NN, Sacks N, Shinde S, Poon JL, Kan H. Clinical, economic, and health-related quality of life outcomes in patients with overweight or obesity in the United States: 2016–2018. Obes Sci Pract. 2024;10(1):e726. 38263999 10.1002/osp4.726 PMC10804324

[12] Soltoft F, Hammer M, Kragh N. The association of body mass index and health-related quality of life in the general population: data from the 2003 Health Survey of England. Qual Life Res. 2009;18(10):1293–9. 19813103 10.1007/s11136-009-9541-8 PMC2788145

[13] Chang HT, Hsu NW, Chen HC, Tsao HM, Lo SS, Chou P. Associations between Body Mass Index and Subjective Health outcomes among older adults: findings from the Yilan Study, Taiwan. Int J Environ Res Public Health. 2018;15(12):2645. 30486260 10.3390/ijerph15122645 PMC6313453

[14] Hainer V, Aldhoon-Hainerova I. Obesity paradox does exist. Diabetes Care. 2013;36(Suppl 2):S276–281. 23882059 10.2337/dcS13-2023 PMC3920805

[15] Yan LL, Daviglus ML, Liu K, Pirzada A, Garside DB, Schiffer L, Dyer AR, Greenland P. BMI and health-related quality of life in adults 65 years and older. Obes Res. 2004;12(1):69–76. 14742844 10.1038/oby.2004.10

[16] Chen S, Ling J, Cheng Y. Physical activity and body mass index were interactively related to health-related quality of life among older adults. Arch Gerontol Geriatr. 2023;104:104833. 36240587 10.1016/j.archger.2022.104833

[17] Minagawa Y, Saito Y. The role of underweight in active life expectancy among older adults in Japan. J Gerontol B Psychol Sci Soc Sci. 2021;76(4):756–65. 32016426 10.1093/geronb/gbaa013

[18] Herman KM, Hopman WM, Vandenkerkhof EG, Rosenberg MW. Physical activity, body mass index, and health-related quality of life in Canadian adults. Med Sci Sports Exerc. 2012;44(4):625–36. 21971297 10.1249/MSS.0b013e31823a90ae

[19] Kim H, Yoon JL, Lee A, Jung Y, Kim MY, Cho JJ, Ju YS. Prognostic effect of body mass index to mortality in Korean older persons. Geriatr Gerontol Int. 2018;18(4):538–46. 29214747 10.1111/ggi.13213

[20] Sach TH, Barton GR, Doherty M, Muir KR, Jenkinson C, Avery AJ. The relationship between body mass index and health-related quality of life: comparing the EQ-5D, EuroQol VAS and SF-6D. Int J Obes (Lond). 2007;31(1):189–96. 16682976 10.1038/sj.ijo.0803365

[21] Visscher TL, Rissanen A, Seidell JC, Heliovaara M, Knekt P, Reunanen A, Aromaa A. Obesity and unhealthy life-years in adult finns: an empirical approach. Arch Intern Med. 2004;164(13):1413–20. 15249350 10.1001/archinte.164.13.1413

[22] Flegal KM, Graubard BI, Williamson DF, Gail MH. Excess deaths associated with underweight, overweight, and obesity. JAMA. 2005;293(15):1861–7. 15840860 10.1001/jama.293.15.1861

[23] Luah XW, Holst-Hansen T, Lübker C. The association between body mass index and health-related quality of life in the 2017 and 2018 health survey of England data: a cross-sectional observational analysis. Diabetes Obes Metabolism. 2024;26(6):2318–28. 10.1111/dom.15546 38499493

[24] Galvin AE, Friedman DB, Hebert JR. Focus on disability-free life expectancy: implications for health-related quality of life. Qual Life Res. 2021;30(8):2187–95. 33733432 10.1007/s11136-021-02809-1 PMC7970769

[25] Corica F, Bianchi G, Corsonello A, Mazzella N, Lattanzio F, Marchesini G. Obesity in the context of aging: quality of life considerations. PharmacoEconomics. 2015;33(7):655–72. 25420750 10.1007/s40273-014-0237-8

[26] Zheng W, McLerran DF, Rolland B, Zhang X, Inoue M, Matsuo K, He J, Gupta PC, Ramadas K, Tsugane S, et al. Association between body-mass index and risk of death in more than 1 million asians. N Engl J Med. 2011;364(8):719–29. 21345101 10.1056/NEJMoa1010679 PMC4008249

[27] Ministry of Government Legislation, Regional Public Health Act. 2023. https://www.law.go.kr/LSW/eng/engLsSc.do?menuId=2&section=lawNm&query=%EC%A7%80%EC%97%AD%EB%B3%B4%EA%B1%B4%EB%B2%95&x=32&y=20#liBgcolor0. Accessed 1 Sep 2023.

[28] Kang YW, Ko YS, Kim YJ, Sung KM, Kim HJ, Choi HY, Sung C, Jeong E. Korea Community Health Survey Data profiles. Osong Public Health Res Perspect. 2015;6(3):211–7. 26430619 10.1016/j.phrp.2015.05.003 PMC4551141

[29] Korea Disease Control and Prevention Agency, Community Health Survey. 2023. https://chs.kdca.go.kr/chs/main.do. Accessed 11 May 2023.

[30] Rabin R, de Charro F. EQ-5D: a measure of health status from the EuroQol Group. Ann Med. 2001;33(5):337–43. 11491192 10.3109/07853890109002087

[31] EQ-5D Korean Valuation Study Using Time Trade Off Method. In. Cheongju-si: Korea Disease Control and Prevention Agency; 2007.

[32] Kim MH, Cho YS, Uhm WS, Kim S, Bae SC. Cross-cultural adaptation and validation of the Korean version of the EQ-5D in patients with rheumatic diseases. Qual Life Res. 2005;14(5):1401–6. 16047514 10.1007/s11136-004-5681-z

[33] Deurenberg P, Deurenberg-Yap M, Guricci S. Asians are different from caucasians and from each other in their body mass index/body fat per cent relationship. Obes Rev. 2002;3(3):141–6. 12164465 10.1046/j.1467-789x.2002.00065.x

[34] Kim BY, Kang SM, Kang JH, Kang SY, Kim KK, Kim KB, Kim B, Kim SJ, Kim YH, Kim JH, et al. 2020 Korean Society for the Study of Obesity Guidelines for the management of obesity in Korea. J Obes Metab Syndr. 2021;30(2):81–92. 34045368 10.7570/jomes21022 PMC8277596

[35] Ministry of Government Legislation, National Basic Living Security Act. 2023. https://www.law.go.kr/LSW/eng/engLsSc.do?menuId=2&section=lawNm&query=NATIONAL+BASIC+LIVING+SECURITY+ACT&x=38&y=24#liBgcolor1. Accessed 1 Sep 2023.

[36] Ministry of Government Legislation, Local Autonomy Act. 2023. https://www.law.go.kr/LSW/eng/engLsSc.do?menuId=2&section=lawNm&query=%EC%A7%80%EB%B0%A9%EC%9E%90%EC%B9%98%EB%B2%95&x=0&y=0#liBgcolor2. Accessed 2 Sep 2023.

[37] Ryu SY, Park J, Choi SW, Han MA. Associations between socio-demographic characteristics and healthy lifestyles in Korean adults: the result of the 2010 Community Health Survey. J Prev Med Public Health. 2014;47(2):113–23. 24744828 10.3961/jpmph.2014.47.2.113 PMC3988282

[38] Heo J, Choi WJ, Ham S, Kang SK, Lee W. Association between breakfast skipping and metabolic outcomes by sex, age, and work status stratification. Nutr Metab (Lond). 2021;18(1):8. 33413444 10.1186/s12986-020-00526-z PMC7788749

[39] Yoon NH, Kwon S. The effects of community environmental factors on obesity among Korean adults: a multilevel analysis. Epidemiol Health. 2014;36:e2014036. 25666167 10.4178/epih/e2014036 PMC4322521

[40] Shiwaku K, Anuurad E, Enkhmaa B, Nogi A, Kitajima K, Shimono K, Yamane Y, Oyunsuren T. Overweight Japanese with body mass indexes of 23.0-24.9 have higher risks for obesity-associated disorders: a comparison of Japanese and mongolians. Int J Obes Relat Metab Disord. 2004;28(1):152–8. 14557832 10.1038/sj.ijo.0802486

[41] Bottone FG Jr., Hawkins K, Musich S, Cheng Y, Ozminkowski RJ, Migliori RJ, Yeh CS. The relationship between body mass index and quality of life in community-living older adults living in the United States. J Nutr Health Aging. 2013;17(6):495–501.23732544 10.1007/s12603-013-0022-y

[42] Selvamani Y, Singh P. Socioeconomic patterns of underweight and its association with self-rated health, cognition and quality of life among older adults in India. PLoS ONE. 2018;13(3):e0193979. 29513768 10.1371/journal.pone.0193979 PMC5841798

[43] Demarest J, Allen R. Body image: gender, ethnic, and age differences. J Soc Psychol. 2000;140(4):465–72. 10981375 10.1080/00224540009600485

[44] Lopez-Garcia E, Banegas Banegas JR, Gutierrez-Fisac JL, Perez-Regadera AG, Ganan LD, Rodriguez-Artalejo F. Relation between body weight and health-related quality of life among the elderly in Spain. Int J Obes Relat Metab Disord. 2003;27(6):701–9. 12833114 10.1038/sj.ijo.0802275

[45] Asgeirsdottir TL. Do body weight and gender shape the work force? The case of Iceland. Econ Hum Biol. 2011;9(2):148–56. 21196135 10.1016/j.ehb.2010.12.001

[46] Halaweh H, Willen C, Grimby-Ekman A, Svantesson U. Physical activity and health-related quality of life among Community Dwelling Elderly. J Clin Med Res. 2015;7(11):845–52. 26491496 10.14740/jocmr2307w PMC4596265

[47] Minet Kinge J, Morris S. Socioeconomic variation in the impact of obesity on health-related quality of life. Soc Sci Med. 2010;71(10):1864–71. 20932623 10.1016/j.socscimed.2010.09.001

[48] Kim GM, Hong MS, Noh W. Factors affecting the health-related quality of life in community-dwelling elderly people. Public Health Nurs. 2018;35(6):482–9. 29947059 10.1111/phn.12530

[49] Wham CA, Teh R, Moyes S, Dyall L, Kepa M, Hayman K, Kerse N. Health and Social Factors Associated with Nutrition Risk: results from Life and living in Advanced Age: a Cohort Study in New Zealand (LiLACS NZ). J Nutr Health Aging. 2015;19(6):637–45.26054500 10.1007/s12603-015-0514-z

[50] Kao YH, Celestin MD Jr., Yu Q, Moody-Thomas S, Jones-Winn K, Tseng TS. Racial and income disparities in Health-related quality of life among smokers with a quit attempt in Louisiana. Med (Kaunas) 2019, 55(2). 10.3390/medicina55020048 PMC6410197 30781893

[51] Guiterrez-Bedmar M, Segui-Gomez M, Gomez-Gracia E, Bes-Rastrollo M, Martinez-Gonzalez MA. Smoking status, changes in smoking status and health-related quality of life: findings from the SUN (Seguimiento Universidad De Navarra) cohort. Int J Environ Res Public Health. 2009;6(1):310–20. 19440285 10.3390/ijerph6010310 PMC2672342

[52] Jing Z, Li J, Wang Y, Yuan Y, Zhao D, Hao W, Yu C, Zhou C. Association of smoking status and health-related quality of life: difference among young, middle-aged, and older adults in Shandong, China. Qual Life Res. 2021;30(2):521–30. 32989682 10.1007/s11136-020-02645-9

[53] Saito I, Okamura T, Fukuhara S, Tanaka T, Suzukamo Y, Okayama A, Ueshima H. A cross-sectional study of alcohol drinking and health-related quality of life among male workers in Japan. J Occup Health. 2005;47(6):496–503. 16369112 10.1539/joh.47.496

[54] Ortolá R, García-Esquinas E, Galán I, Rodríguez-Artalejo F. Patterns of alcohol consumption and health-related quality of life in older adults. Drug Alcohol Depend. 2016;159:166–73. 26748410 10.1016/j.drugalcdep.2015.12.012

[55] Powers JR, Young AF. Longitudinal analysis of alcohol consumption and health of middle-aged women in Australia. Addiction. 2008;103(3):424–32. 18269363 10.1111/j.1360-0443.2007.02101.x

[56] Winter JE, MacInnis RJ, Wattanapenpaiboon N, Nowson CA. BMI and all-cause mortality in older adults: a meta-analysis. Am J Clin Nutr. 2014;99(4):875–90. 24452240 10.3945/ajcn.113.068122

[57] Gill LE, Bartels SJ, Batsis JA. Weight Management in older adults. Curr Obes Rep. 2015;4(3):379–88. 26627496 10.1007/s13679-015-0161-z PMC5387759

[58] Connor Gorber S, Tremblay M, Moher D, Gorber B. A comparison of direct vs. self-report measures for assessing height, weight and body mass index: a systematic review. Obes Rev. 2007;8(4):307–26. 17578381 10.1111/j.1467-789X.2007.00347.x

[59] Wu M, Brazier JE, Kearns B, Relton C, Smith C, Cooper CL. Examining the impact of 11 long-standing health conditions on health-related quality of life using the EQ-5D in a general population sample. Eur J Health Econ. 2015;16(2):141–51. 24408476 10.1007/s10198-013-0559-z PMC4339694

